# Interaction Profile of Diphenyl Diselenide with Pharmacologically Significant Thiols

**DOI:** 10.3390/molecules171012287

**Published:** 2012-10-19

**Authors:** Waseem Hassan, Joao Batista Teixeira Rocha

**Affiliations:** 1 Department of Chemistry, Center of Natural and Exact Sciences, Federal University of Santa Maria, CEP 97105-900 Santa Maria, RS, Brazil; 2 Institute of Chemical Sciences, University of Peshawar, Peshawar 25120, Khyber Pakhtunkhwa, Pakistan

**Keywords:** pH, diphenyl diselenide, thiol oxidation

## Abstract

Diphenyl diselenide has shown interesting biological activities in various free-radical-induced damage models and can be considered as a potential candidate drug against oxidative stress. Apart from its anti-oxidant activity, this compound can oxidize various thiols. However there are no detailed studies in the literature about the thiol oxidase-like activity of this compound against biologically significant mono and di-thiols with respect to various pH conditions. Keeping in mind the scarcity of data in this area of organochalcogen chemistry, we report for the first time the kinetics of thiol oxidation by diphenyl diselenide, which was carried out in a commonly used phosphate buffer, not only at physiological pH, but also at a number of acidic values. The relative reactivities of the different thiols with diphenyl diselenide were independent of the pKa of the thiol group, such that at pH 7.4, cysteine and dithiothreitol were the most reactive, while 2,3-dimercapto-1-propanesulfonic acid and glutathione were weakly reactive and extremely low reactivity was observed with dimercaptosuccinic acid. Rate of oxidation was dependent on the pH of the incubation medium. The results obtained will help us in the design of rational strategies for the safe pharmacological use of diphenyl diselenide.

## 1. Introduction

Thiol-containing compounds play an important role in protecting biological systems against oxidative injury. There is also increasing evidence for thiol involvement in metabolic regulation, signal transduction and regulation of gene expression [1]. Organoselenium compounds, including diphenyl diselenide (DPDS) are known to possess neuroprotective, anticancer, anti-nociceptive, anti-hypercolesterolemic, pro-apoptotic and anti-inflammatory activities in different experimental models of human pathologies [2,3,4,5,6,7,8]. Organoselenium compounds can interact directly with low molecular thiols, oxidizing them to disulfides [9]. Similarly, reduced cysteinyl residues from proteins can also react with these compounds, which may cause, in the case of the enzymes, the loss of their catalytic activity [10,11,12,13]. The mechanism(s) underlying either the toxic or protective effect of organochalcogens are not completely understood, but certainly involve the reaction of chalcogenides with endogenous thiols [14,15]. 

Physiological thiols vary substantially in their reactivity, and on this basis, thiol groups would be preferred cellular targets for various oxidants. However the relative reactivity of different physiological thiols with DPDS has not been established. The formation of stable selenolate (RSe^−1^) ions [16,17,18,19,20,21] can increase the reducing properties of these moieties on the organochalcogenides and hypothetically can increase their antioxidant properties. Based on this hypothesis we can assume that low pH can stabilize the selenolate (RSe^−1^) ion by forming a selenol (–SeH) molecule and hypothetically this will reduce the rate of thiol oxidation. However, there is no data in the literature supporting this assumption. In fact, the thiol oxidase-like activity of diselenides have been studied only at physiological pH values and no detailed comparative study about the effect of pH on the interaction of thiols and DPDS_, _the simplest of the aryl diselenide, is available in the literature. 

Thus, it is highly relevant to develop an insight into the interaction profile of this interesting organoselenium compound. The goal of our study was to investigate whether the thiol oxidase-like activity of DPDS could be affected by changes in pH. Specifically we determined the influence of pH on the reactivity of DPDS towards different thiol-containing molecules. In this paper we have studied the reactivity of glutathione (GSH) cysteine (CYS), 2,3-dimercapto-1-propanesulfonic acid (DMPS), dimercaptosuccinic acid (DMSA) and dithiothreitol (DTT) with DPDS and reported influence of pH on the reactivity of DPDS with these biologically significant thiols. 

## 2. Results and Discussion

Oxidation of thiols by DPDS in phosphate buffer was followed by measuring the decrease in thiol concentration as a function of time. Loss of thiol was monitored by measuring the decrease in the absorbance at 412 nm due to the 5-thio-2-nitrobenzoate dianion (TNB) disulfide exchange reaction. The structures of DTT, GSH, CYS, DMPS and DMSA are shown in Table 1, while the respective rates constant are shown in Table 2. At physiological pH (7.4) CYS has higher rate constant as compared to GSH and DTT. These results can be explained on the basis of the extent of ionization of a thiol and the intrinsic nucleophilicity of the corresponding thiolate anion. Literature has demonstrated that the lower the pKa of a thiol the lower will be the nucleophilicity of the thiolate, but the higher the relative concentration of thiolate anions will be [22,23]. The pKa values of CYS, GSH and DTT are 8.3, 8.8 and 9.1 respectively; this means that at physiological pH the CYS with the lowest pKa value would have comparatively higher thiolate ion concentration. The higher thiolate ion concentration is responsible for the observed higher rate of oxidation for the cysteine. 

**Table 1:** The chemical structures of DPDS and thiols.

Compound	Structure
**DPDS**	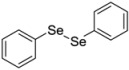
**CYS**	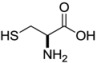
**GSH**	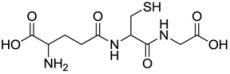
**DTT**	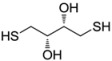
**DMPS**	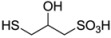
**DMSA**	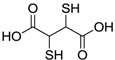

**Table 2:** Second order rate constants of reaction of the different thiols in the absence or presence of DPDS at physiological pH (7.4).

Thiol	*k*_2_ [M^−1^s^−1^]	
	DPDS	Ethanol
CYS	1059 ± 38	967 ± 13
DMPS	172 ± 12	160 ± 19
DMSA	116 ± 17	97 ± 13
DTT	970 ± 22	492 ± 12
GSH	163 ± 8	113 ± 11

However in strong contradiction with the above explanation we can see from Table 2 that GSH has a lower rate constant than DTT at pH 7.4, both in the absence and presence of DPDS. Based on simple pKa values and ultimate thiolate ion concentration phenomenon GSH oxidation should be faster than that of DTT. A plausible explanation could be that the GSH molecule is sterically more hindered than DTT. By looking at the DTT structure one can observe that it has two proximal –SH groups which not only facilitate auto-oxidation but may also promote DPDS-induced oxidation process. This data is in strong agreement with our previous report that ð-ALA-D (a –SH containing enzyme) from plants, in marked contrast to the enzyme from rats, was not inhibited by diphenyl diselenide. It is worthy to note that the plant enzyme has no cysteinyl residues in close proximity, as observed in the active site of the mammalian enzyme [14]. We have also obtained strong evidence that DTT is a significantly better substrate than CYS or GSH for the oxidation catalyzed by diorganoyl diselenides and tellurides [14]. 

It is apparent from the Table 2 that DMSA and DMPS have relatively lower rate constants. The observed reactivity could also be attributed to extreme steric hindrance provided by the close carboxyl groups. The lower nucleophilicity of these two thiols and its role cannot be neglected in the DPDS induced oxidation. 

**Figure 1:**
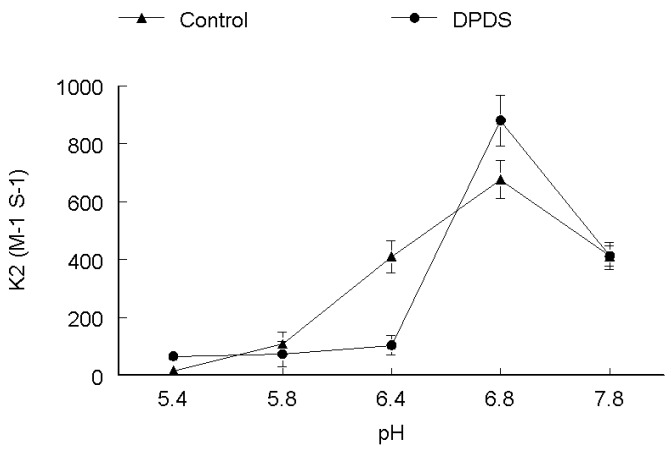
pH-dependence of the second order rate constant of the reaction of DPDS (**●**) and ethanol (**▲**) with cysteine. ***k*_2_ (M^−1^ s^−1^)**

In order to elucidate the effect of pH on the rate of oxidation, five to seven runs were performed at different pH ranges (5.4–7.8). Two different sets of experiments were performed. In the first set the thiol concentration was kept constant and DPDS concentration was varied, while, in the second set DPDS concentration was kept constant and thiol concentration was varied. Rate constant for cysteine oxidation tended to increase as function of pH and on whether it was determined in the presence or absence of DPDS. Rate constant for cysteine oxidation increased about 3–4 times as pH increased from 5.4 to 7.8 as shown in Figure 1. 

**Figure 2:**
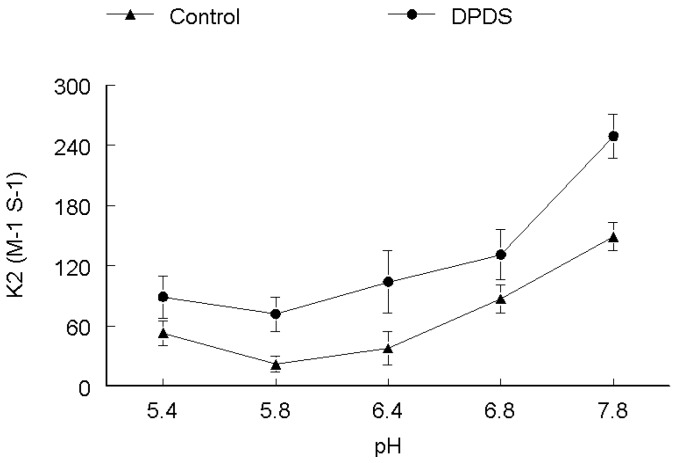
pH-dependence of the second order rate constant of the reaction of DPDS (**●**) and ethanol (**▲**) with DMPS. ***k*_2_ (M^−1^ s^−1^)**

**Figure 3:**
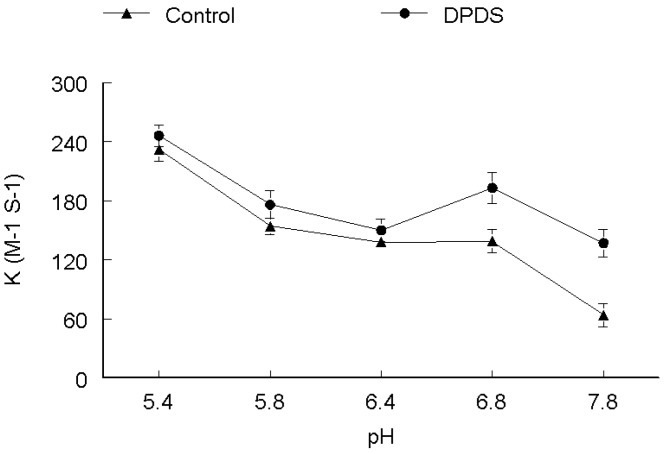
pH-dependence of the second order rate constant of the reaction of DPDS (**●**) and ethanol (**▲**) with DMSA. ***k*_2_ (M^−1^ s^−1^)**

**Figure 4:**
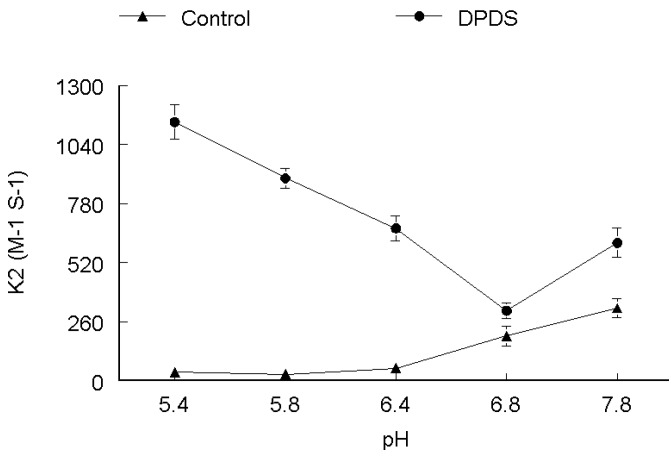
pH-dependence of the second order rate constant of the reaction of DPDS (**●**) and ethanol (**▲**) with DTT. ***k*_2_ (M^−1^ s^−1^)**

The rate of DMPS oxidation increased with increasing pH as depicted in Figure 2. There was no difference in the trend of reactivities both in the absence and presence of DPDS. The rate was lower at low pH, *i.e.*, 5.4. However, at higher pH values from 6.4–7.8 the rate of oxidation increased both in the presence and absence of DPDS, reaching a maximum at pH 7.8, which is about three times higher as compared with pH 5.4. The rate constant of DMPS oxidation was increased about 50% at all pH values in the presence of DPDS. It is apparent from Figure 3 that pH changed the rate of DMSA oxidation both in the absence and presence of DPDS. The rate of oxidation (both in the absence and presence of DPDS) was higher at pH 5.4. In the absence of DPDS, the rate of reaction decreased from pH 5.4 to 7.8 in a linear fashion. In the presence of DPDS, the rate decreased linearly up to pH 6.4 and then increased about 30% at pH 6.8 and then decrease again at pH 7.8 to values similar to that observed at pH 6.4. In the absence of DPDS, the rate of DTT oxidation was extremely low between pH values of 5.4 to 6.4 and then increase in a linear way between 6.8 and 7.8. In the presence of DPDS, the rate of DTT oxidation was higher at more acid pH values and decreased linearly from pH 5.4 to 6.8 and then slowly increased at pH 7.8 as shown in Figure 4. 

**Figure 5:**
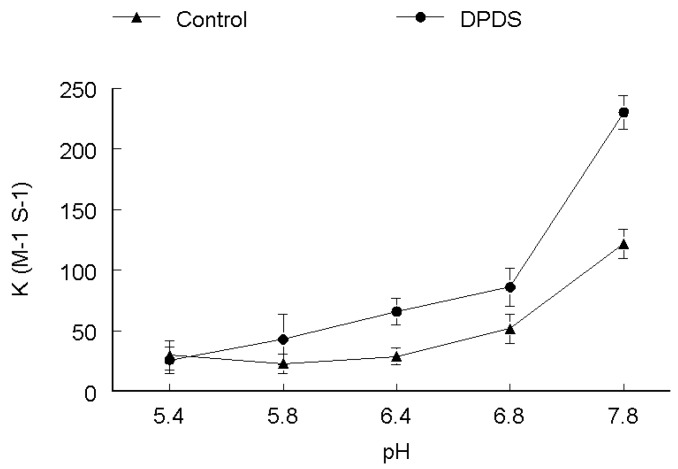
pH-dependence of the second order rate constant of the reaction of DPDS (**●**) and ethanol (**▲**) with GSH. ***k*_2_ (M^−1^ s^−1^)**

In the absence and presence of DPDS, the rate of GSH oxidation increased as the pH increased from 5.4 to 7.8 however, the increase was slightly faster in the presence of DPDS (Figure 5). At pH 7.8 the rate of GSH oxidation was about 2-times higher in the presence of DPDS.

The effect of pH on rate of reaction for CYS (Figure 1), DMPS (Figure 2) and GSH (Figure 5) revealed that these thiols are more reactive when ionized, which is observable by the gradual increase of the rate constant with increasing pH, both in the absence and presence of DPDS. This indicates that the deprotonated form of these thiols is the active species*.* Our study further revealed that the apparent rate constants for the reaction of DPDS with DMSA and DTT were higher at low pH, as shown in Figure 3 and Figure 4, respectively. The question of higher reactivity at lower pH for carboxylic acid-containing molecules may be related to a decrease in the reactivity of the negative charges as pH lowers, *i.e.*, the steric hindrance of the charges diminishes as pH falls, while in the case of DTT we propose that the reaction may be acid catalyzed.

**Scheme 1:**
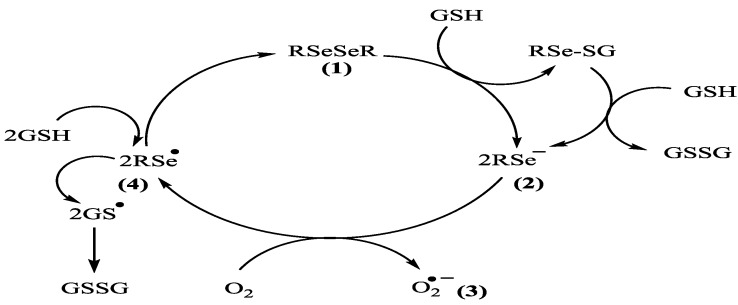
Catalytic oxidation cycle of diphenyl diselenide.

**Scheme 2:**
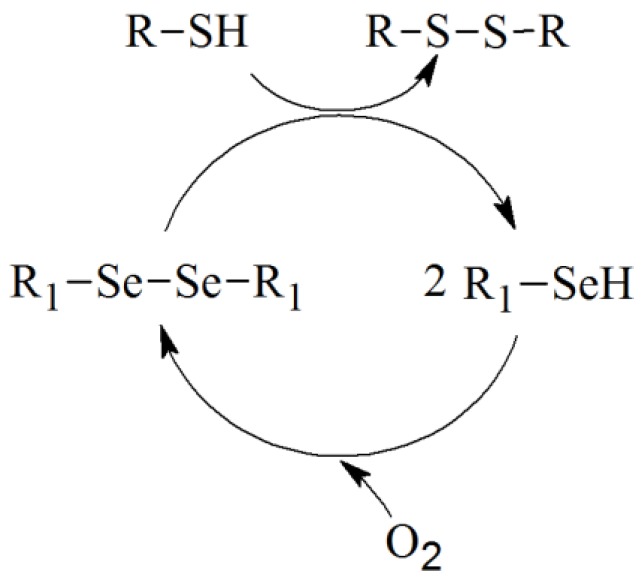
Proposed reactions for thiols oxidation by DPDS.

There have been a number of reports suggesting that organoselenium compounds such as selenocystine and a variety of diorganodiselenides can also react with thiols such as cysteine, dithiothreitol, and reduced glutathione to produce selenocysteine, selenols, and disulfides [24]. In this regard a proposed catalytic cycle for diphenyl diselenide/thiol interaction has been described by our laboratory (Scheme 1) [25]. Accordingly, we realized that the first step in Scheme 1 involves the reaction of diphenyl diselenide (**1**) with thiol, which give an unstable RSe^−1^ type intermediate **2**. The catalytic mechanism produces superoxide **3** as a one step electron transfer from RSe^-1^ to dioxygen and further hydrogen peroxide with the formation of RSe^•^ (radical) **4** which further regenerates diphenyl diselenide (**1**). Our group [25] has shown that the (PhSe)_2_, can accelerate the rate of thiol oxidation even in the absence of peroxide by catalyzing the reactions shown in Scheme 2. 

Herein we provided for the first time the rate constants for various thiols, this will help us to understand the molecular basis of diphenyl diselenide induce antioxidant and toxicological profile.

## 3. Experimental

### 3.1. Chemicals

Dithiothreitol (DTT), 5,5'-dithio-bis(2-nitrobenzoic acid) (DTNB), glutathione (GSH), cysteine (CYS), 2,3-dimercapto-1-propanesulfonic acid (DMPS), dimercaptosuccinic acid (DMSA) (Table 1) were purchased from Merck (Darmstadt, Germany). All other chemicals were of analytical grade and obtained from standard commercial suppliers.

### 3.2. DPDS Synthesis and Preparation

DPDS (Table 1) was synthesized according to literature method [26] and was dissolved in ethanol. Analysis of the ^1^H-NMR and ^13^C-NMR spectra showed that the compound obtained presented analytical and spectroscopic data in full agreement with its assigned structure. The chemical purity of DPDS (99.9%) was determined by GC/HPLC. Solutions of DPDS were prepared in ethanol a few minutes (5–10 min) before the experiments. The solutions are stable for the period utilized in the experimental protocols performed in this study. 

### 3.3. Preparation of Buffers

All buffers were prepared at room temperature with constant ionic strength. Buffer solutions were maintained at 4 °C until the initiation of the experiment. Direct measurement of pH values in the tubes at higher temperature *i.e.*, (37 °C) verified that actual pH values were typically within ±0.05. The final buffer concentration in all experiments was 50 mM.

### 3.4. The Rate of Thiol Oxidation

Thiol oxidation was evaluated by measuring the disappearance of –SH groups according to the method of Ellman *et al.* [27]. Incubation at 37 °C was initiated by adding specific concentration (0.1, 0.5, 1.0 and 2.0 mM) of GSH, CYS, DMPS, DMSA and DTT to a medium containing 50 mM sodium phosphate buffer at different pH values. The pH of the buffer solutions ranged from 5.4 to 7.8 (5.4, 5.8, 6.4, 6.8, 7.4 and 7.8) and were checked by an Orion Research digital pH/millivolt meter (Model 611). Aliquots of 60 μL were withdrawn at different time intervals and was used to determine the amount of –SH groups at 412 nm after reaction with 5,5'-dithio-bis(2-nitrobenzoic acid) (DTNB). Spectral measurements were performed by using Hitachi 2001 spectrophotometer. Dependence of the rate of oxidation on pH was investigated in sodium phosphate buffer solutions at constant ionic strength. The reaction between DPDS and thiol was further studied in order to determine the reaction order. Its order in each thiol was determined by carrying out the reaction at a constant DPDS concentration (20 µM) and varying concentrations of thiol (0.1–2.0 mM). Progress of the reaction was followed by periodic titration of the residual free thiol with DTNB. In a second set of experiments, the reaction between thiol and DPDS was followed at a constant thiol concentration (1 mM) and varying DPDS concentrations (2.5–40 µM).

## 4. Conclusions

DPDS has shown numerous protective effects in a variety of free radical induced oxidation models. Herein we proved that this interesting organoselenium compounds can oxidize biologically significant thiols. These effects may be linked to the pro-oxidant activity of DPDS. Design and synthesis of organoselenium compounds with high thiol peroxidase-like activity and low thiol oxidase-like activity has been a difficult task. Further studies are needed to decode the border between toxicological and pharmacological effects of DPDS which may affect cellular functions.

## References

[1] Kettle A.J., Winterbourn C.C. (1997). A key regulator of neutrophil oxidant production. Redox. Rep..

[2] Nogueira C.W., Rocha J.B. (2011). Toxicology and pharmacology of selenium: Emphasis on synthetic organoselenium compounds. Arch. Toxicol..

[3] de Freitas A.S., Prestes A.S., Wagner C., Sudati J.H., Alves D., Porciúncula L.O., Kade I.J., Rocha J.B.T. (2010). Reduction of diphenyl diselenide and analogs by mammalian thioredoxin reductase is independent of their gluthathione peroxidase-like activity: A possible novel pathway for their antioxidant activity. Molecules.

[4] Puntel R.L., Roos D.H., Folmer V., Nogueira C.W., Galina A., Aschner M., Rocha J.B.T. (2010). Mitochondrial dysfunction induced by different organochalchogens is mediated by thiol oxidation and is not dependent of the classical mitochondrial permeability transition pore opening. Toxicol. Sci..

[5] Rupil L.L., de Bem A.F., Roth G. (2012). Diphenyl diselenide-modulation of macrophage activation: Down-regulation of classical and alternative activation markers. Innate Immun..

[6] Posser T., de Paula M.T., Franco J.L., Leal R.B., Rocha J.B.T. (2010). Diphenyl diselenide induces apoptotic cell death and modulates ERK1/2 phosphorylation in human neuroblastoma SH-SY5Y cells. Arch. Toxicol..

[7] de Bem A.F., Farina M., Portella R.L., Nogueira C.W., Dinis T.C.P., Laranjinha J.A.N., Almeida L.M., Rocha J.B.T. (2008). Diphenyl diselenide, a simple glutathione peroxidase mimetic, inhibits human LDL oxidation *in vitro*. Atherosclerosis.

[8] Corte C.L.D., Soares F.A.A., Aschner M., Rocha J.B.T. (2012). Diphenyl diselenide prevents methylmercury-induced mitochondrial dysfunction in rat liver slices. Tetrahedron.

[9] Goeger D.E., Ganther H.E. (1994). Oxidation of dimethyl selenide to dimethyl selenoxide by microsomes from rat liver and flavin-containing monooxygenase from pig liver. Arch. Biochem. Biophys..

[10] Björnstedt M., Odlander B., Kuprin S. (1996). Selenite incubated with NADPH and mammalian thioredoxin reductase yelds selenide, which inhibits lipoxygenase and changes the electron spin resonance spectrum of the active site iron. Biochemistry.

[11] Park H.S., Park E., Kim M.S., Ahn K., Kim I.Y., Choi E.J. (2000). Selenite inhibits the c-Jun N-terminal kinase/stress-activated protein kinase (JNK/SAPK) through a thiol redox mechanism. J. Biol. Chem..

[12] Gupta N., Porter T.D. (2001). Inhibition of human squalene monooxygenase by selenium compounds. J. Biochem. Mol. Toxicol..

[13] Rocha J.B.T., Saraiva R.A., Garcia S.C., Gravina F.S., Nogueira C.W. (2012). Aminolevulinate dehydratase (δ-ALA-D) as marker protein of intoxication with metals and other pro-oxidant situations. Toxicol. Res..

[14] Maciel E.N., Bolzan R.C., Braga A.L., Rocha J.B.T. (2000). Diphenyl diselenide and diphenyl ditelluride differentially affect d-aminolevulinate dehydratase from liver, kidney, and brain of mice. J. Biochem. Mol. Toxicol..

[15] Daiber A., Zhou M., Bachscmid M., Ullrich V. (2000). Ebselen as a peroxynitrite scavenger *in vitro* and *ex vivo*. Biochem. Pharmacol..

[16] Santi C., Santoro S., Battistelli B., Testaferri L., Tiecco M. (2008). Preparation of the First Bench-Stable Phenyl Selenolate: An Interesting “On Water” Nucleophilic Reagent. Eur. J. Org. Chem..

[17] Battistelli B., Lorenzo T., Tiecco M., Santi C. (2011). On-Water” Michael-Type Addition Reactions Promoted by PhSeZnCl. Eur. J. Org. Chem..

[18] Santoro S., Battistelli B., Testaferri L., Tiecco M., Santi C. (2009). Vinylic Substitutions Promoted by PhSeZnCl: Synthetic and Theoretical Investigations. Eur. J. Org. Chem..

[19] Santi C., Battistelli B., Testaferri L., Tiecco M. (2012). On water preparation of phenylselenoesters. Green Chem..

[20] Santi C., Santoro S., Testaferri L., Tiecco M. (2008). Simple zinc-mediated preparation of selenols. ChemInform.

[21] Tidei C., Piroddi M., Galli F., Santi C. (2012). Oxidation of thiols promoted by PhSeZnCl. Tetrahedron Lett..

[22] Jocelyn P.C. (1972). The Biochemistry of the SH Group.

[23] Lindley H. (1960). Study of the Kinetics of the Reaction between. Thiol Compounds and Chloroacetamide. J. Biochem..

[24] Burk R.F. (1994). Selenium in Biology and Human Health.

[25] Nogueira C.W., Zeni G., Rocha J.B.T. (2004). Organoselenium and organotellurium compounds: Toxicology and pharmacology. Chem. Rev..

[26] Paulmier C. (1986). Selenium Reagents and Intermediates in Organic Synthesis.

[27] Ellman G.D. (1959). Assay for lipid peroxides in animal tissues by thiobarbituric acid reaction. Tissue sulphydryl groups. Arch. Biochem. Biophys..

